# Identification and validation of biomarkers related to Th1 cell infiltration in neuropathic pain

**DOI:** 10.1186/s12950-023-00343-0

**Published:** 2023-06-01

**Authors:** Xiangsheng Zhang, Jiurong Cheng, Yingdong Deng, Caiyun Guo, Yu Cao, Suo Wang, Chenxi Zhou, Ziqiang Lin, Simin Tang, Jun Zhou

**Affiliations:** The Third Affiliated Hospital of Southern Medical University, Guangzhou, China

**Keywords:** Biomarkers, Macrophages, Th1 cells, Neuroinflammation, Neuropathic pain, Abca1

## Abstract

Neuropathic pain (NP) is a widespread chronic pain with a prevalence of 6.9–10% in the general population, severely affecting patients’ physical and mental health. Accumulating evidence indicated that the immune environment is an essential factor causing NP. However, the mechanism is unclear. This study attempted to analyze NP-related immune infiltration patterns. We downloaded the expression profiles from the Gene Expression Omnibus (GEO) database. The novel method of single-sample gene set enrichment analysis (ssGSEA) algorithm and weighted gene co-expression network analysis (WGCNA) was applied to identify immune-related genes and verified in vitro and in vivo experiments. The spared nerve injury (SNI) group was closely related to type1 T helper cells (Th1 cells), and two key genes (*Abca1* and *Fyb*) positively correlated with Th1 cell infiltration. At the single-cell level, *Abca1* and *Fyb* were significantly expressed in macrophages. In addition, we verified that Abca1 could affect the function of macrophages. Finally, we hypothesized that Abca1 is involved in the infiltration of Th1 cells into dorsal root ganglion (DRG) tissues and induces NP via immunoinflammatory response. Hence, the present study aimed to elucidate the correlation between NP and neuroinflammation and identify a new therapeutic target for treating NP.

## Introduction

Neuropathic pain (NP) was redefined as pain caused by a lesion or disease of the somatosensory nervous system by The International Association for the Study of Pain (IASP) in 2011 [1]. The main signs and symptoms include spontaneous pain, allodynia, and hyperalgesia [2]. The prevalence of NP is about 6.9–10% worldwide [3], but the diverse etiology, complex pathogenesis, and variable clinical presentation have no effect on the clinical treatment for NP. Thus, understanding the pathogenesis of NP is an urgent requirement to explore the therapeutic targets and develop the targeted drugs [4].

Previous studies on NP have focused on the neuronal changes and the role of peripheral and central sensitization after nerve injury. Accumulating evidence suggested that immune cells and related cytokines are closely associated to NP [5–7]. After peripheral nerve injury, the activation and migration of immune cells and the release of immunologically active substances stimulate the immune response or inflammatory damage of the nervous system, affect the excitability of neurons and the synaptic connection between neurons, and promote the occurrence and development of NP [5, 8].

Some studies illustrated that the interaction between neurons and non-neurons in the spinal dorsal horn is a vital factor that induces and maintains NP after peripheral nerve injury [8, 9]. Furthermore, Costigan et al*.* [10] demonstrated that T cells infiltration and activation in the spinal dorsal horn after peripheral nerve injury contributed to neuropathic hypersensitivity, especially high-level Th1 cells in adults. However, the contribution of non-neuronal cells in the DRG has been studied only slightly. Recently, Liu et al. [11]. demonstrated that macrophages in mice DRG induce and maintain NP. The study also confirmed the interaction between sensory neurons and macrophages, revealing the potential peripheral DRG targets for NP therapy. Therefore, exploring the infiltration pattern of immune cells in the DRG provides a new vision for studying the NP caused by peripheral nerve injury.

Various tools have been developed to search for immune-related biomarkers since the rapid development of bioinformatics [12] but have been rarely used in NP research. In this study, we first applied the single sample gene set enrichment analysis (ssGSEA) algorithm to evaluate the difference in immune cell content in DRG tissues and found higher levels of Th1 cells infiltration in the spinal nerve injury (SNI) group compared to the control (Con) group. Subsequently, the module genes associated with Th1 cells infiltration were identified using weighted gene co-expression network analysis (WGCNA) [13], the module genes most associated with Th1 cells infiltration were identified. Next, the expression characteristics of crucial genes were analyzed at the single-cell level. Finally, the immune-related key genes were validated, and the potential mechanism of *Abca1* was explored in the occurrence and development of NP to provide critical guidance in developing effective immunotherapy strategies.

## Results

### Selecting and preprocessing data

**Fig. 1:**
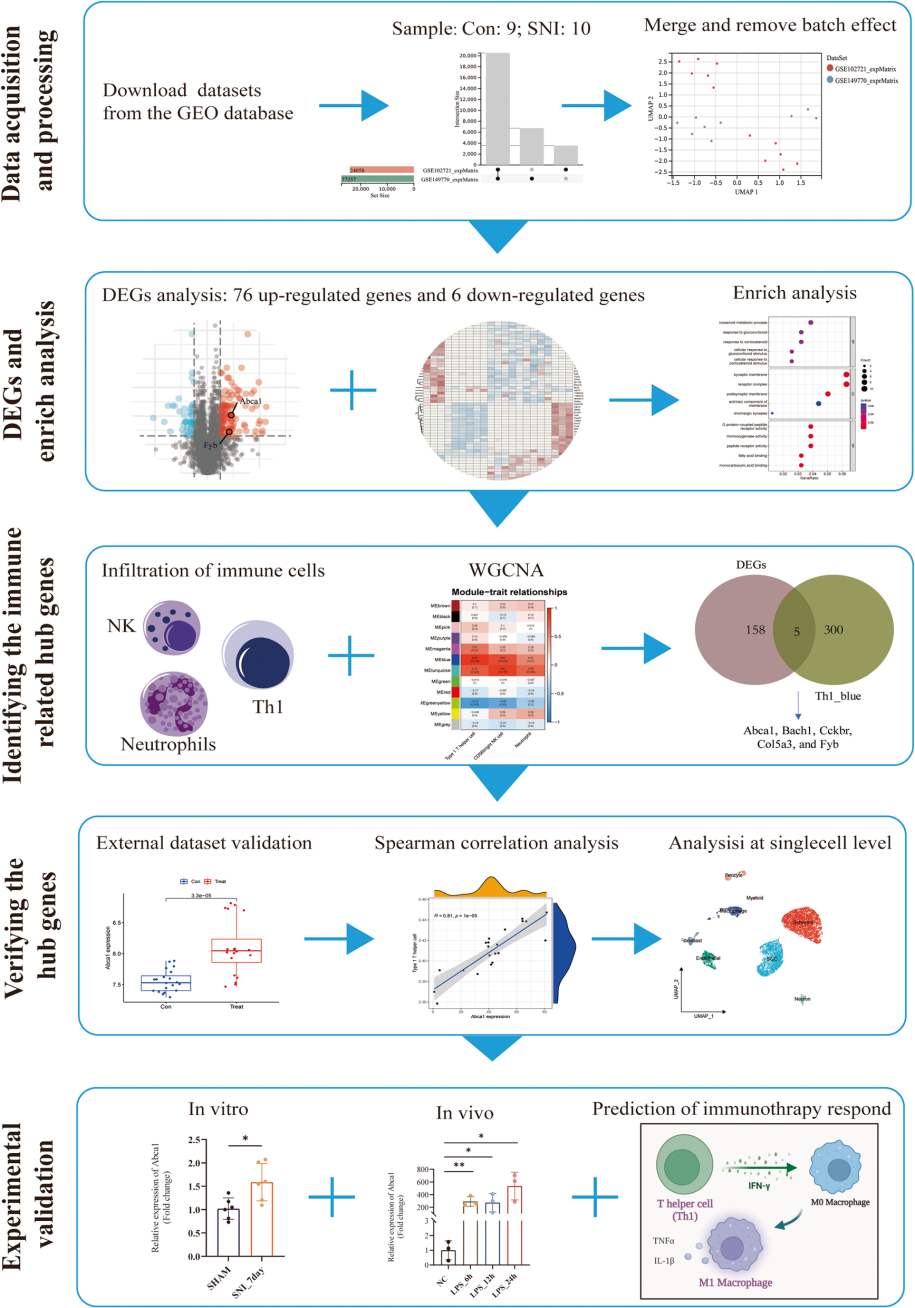
Flow chart of the analysis step in this study. The datasets were downloaded from the GEO database and the differentially expressed genes (DEGs) that were significantly correlated with NP were screened. Enrichment analysis was performed to describe the function of DEGs. ssGSEA algorithm was used to identify significantly different immune cells. WGCNA package was applied to screen Hub genes related to immune cells. Then, Hub genes were verified through external datasets, and spearman was used to analyze the correlation between Hub genes and immune cells. Next, the expression patterns of these Hub genes were detected at the single-cell level. Finally, the expression level of the Hub gene and its related functions will be verified by in vitro and in vivo experiments

**Fig. 2:**
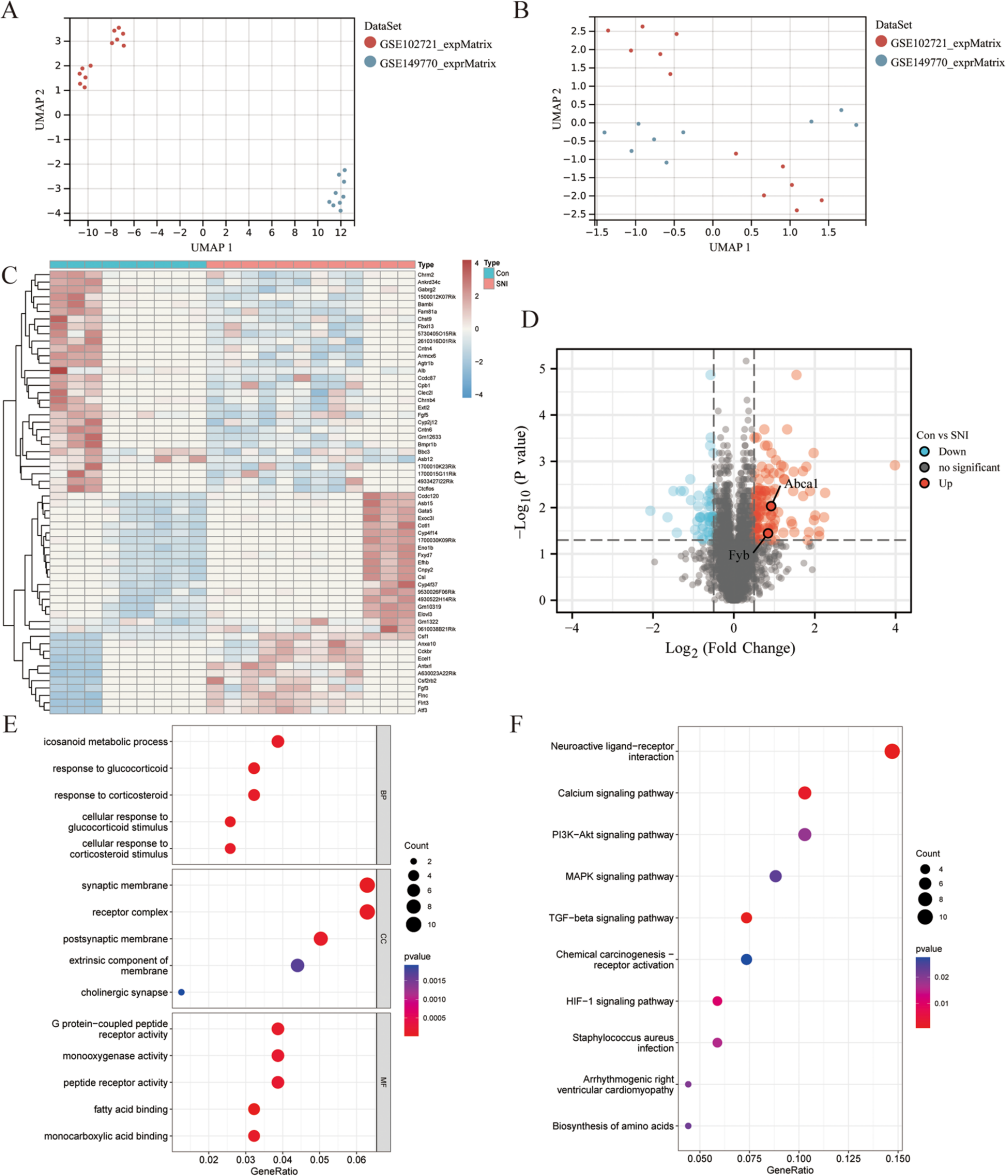
The differentially expressed genes (DEGs) in Dorsal root ganglion (DRG) tissues and enriched analysis. **A** UMAP diagram, the sample distribution of each dataset before the batch effect is removed, and each point represents a sample; (**B**) the sample distribution of each dataset after removing the batch effect; (**C**) Heat map of DEGs, the color represents expression level, the higher expression level, the darker the color (red for up-regulation and blue for down-regulation). The tree cluster on the left represents significant clustering results in different samples; (**D**) Volcano map of DEGs, red represents significantly up-regulated genes, blue represents significantly down-regulated genes, and gray represents genes with no significant differences. **E** Gene Ontology (GO) enrichment analysis bubble diagram, the ordinate is the name of GO and the abscissa is the proportion of genes. The circle size represents the count of DEGs enriched in the GO (the larger the circle, the more DEGs are enriched), and the color represents enrichment significance (the redder the color is, the more significant the DEGs are enriched on this GO). GO analysis includes biological processes (BP), cellular components (CC) and molecular functions (MF); (**F**) Kyoto Encyclopedia of Genes and Genomes (KEGG) enrichment analysis bubble diagram: the circle size represents the count of DEGs enriched to the pathway, the color represents significance

The flowchart of analysis steps is shown in Fig. 1. All data were downloaded from the GEO database and sorted in Table 1. First, we merged GSE102721 and GSE149770 datasets to obtain 9 Con and 10 SNI samples. Then, the batch effect between the data was eliminated. The clustering distribution was uniform after the normalization of the dataset, indicating data reliability (Fig. 2A and B).

**Table 1:** The enrolled datasets in the current study

*Datasets*	Experiment type	Platform	Con/Treat
GSE102721	Expression profiling by high throughput sequencing	GPL21103 Illumina	3/9
GSE149770	Expression profiling by high throughput sequencing	GPL17021 Illumina	6/3
GSE24982	Expression profiling by array	GPL1355 Affymetrix	20/20
GSE174430	Expression profiling by high throughput sequencing	GPL28457 Illumina	1/2

GSE102721 and GSE149770 was used for subsequent analysis, GSE24982 and GSE134003 datasets were used for validation at the whole transcriptome and single cell levels

### Screening for DEGs and enrichment analysis

A total of 163 DEGs (114 upregulated and 49 downregulated) were identified between the Con and SNI groups from the merged gene expression matrix, as shown in the volcano map and heat map (Figs. 2C and D). Figures 2E and F illustrated the results of the functional correlation analysis of GO and KEGG. The results of GO analysis suggested that DEGs are involved in three cellular functions: biological processes (BP), cell components (CC), and molecular function (MF) (Fig. 2E). The main changes in BP were the metabolism and activation of amino acids, such as icosanoid metabolic process, response to glucocorticoid, and response to corticosteroid. The primary variations in CC involved synaptic membrane, receptor complex, and postsynaptic membrane. The significant changes in MF included G protein-coupled peptide receptor activity, monooxygenase activity, peptide receptor activity, and fatty acid binding. KEGG pathway analysis results (Fig. 2F) indicated that DEGs are significantly enriched in neuroactive ligand-receptor interaction and calcium, PI3K-Akt, MAPK, and TGF-beta signaling pathways.

### Screening of significantly different immune cells

**Fig. 3:**
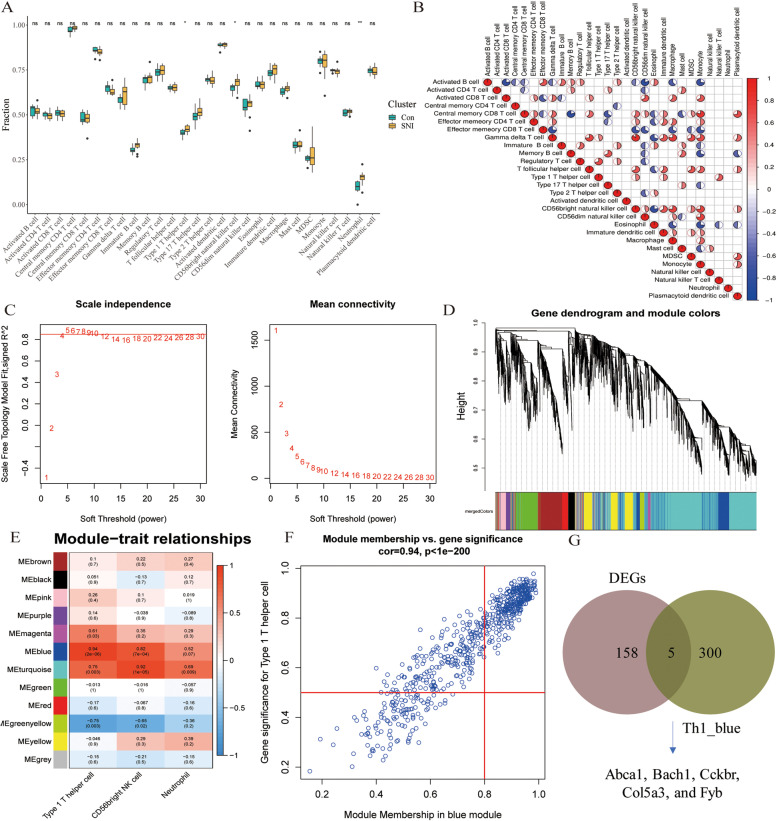
Immune infiltration landscape in DRG tissue and screened Hub genes related to immune traits by WGCNA. **A** Box chart, the difference of immune cell infiltration between the control group and the SNI group (symbol "*", "**", and "ns" respectively represent *p*-value < 0.05, *p*-value < 0.01, and non-significance.); (**B**) Heat map of the correlation of 28 types immune cells, the pie chart will be displayed only when the correlation *P* < 0.05, color represents correlation, color depth represents correlation significance. The darker the red represents the more significant the positive correlation, the darker the blue represents the more significant the negative correlation; (**C**) Network topology analysis under various soft threshold power. When the power value was 4, the independence degree was ≥ 0.85. (On the left: The X-axis shows the soft threshold power. The Y-axis reflects the fit index of the scaled-free topological model. On the right: The X-axis shows the soft threshold power. The Y-axis represents average connectivity (degree)); (**D**) The clustering tree and co-expression network of co-representation network modules are constructed based on 1-TOM matrix; (**E**) Correlation of modules (column) with immune traits (row). Red represents positive correlation, blue, negative correlation. Each unit includes correlation coefficient and *p* value. **F** Scatter plot showed the correlation between gene significance (GS > 0.5) and module membership (MM > 0.8). **G** Venn diagram, the intersection genes between module genes and DEGs

The ssGSEA algorithm was used to analyze the specific immune cell types infiltrated into DRG tissues and elucidate their immune microenvironment. The boxplot showed differences in immune cell infiltration in DRG tissues (Fig. 3A). Compared to the Con group, the infiltration level of type 1 T helper (Th1) cells (*P* < 0.05), CD56bright natural killer (NK) cells (*P* < 0.05), and neutrophils (*P* < 0.01) were higher in the SNI group. Therefore, we defined these three types of significantly different immune cells as the trait data analyzed by WGCNA. In addition, Fig. 3B shows the correlation between 28 immune cells. Correlation heat maps showed that Th1 cells were positively correlated with gamma delta T cells and regulatory T cells. CD56bright NK cells were positively associated with central memory CD8 T cells, gamma delta T cells, and Tfh cells. A negative correlation was established between the activated B cells and effector memory CD8 T cells.

### Identifying immune-cell infiltration-related genes

During WGCNA analysis, we chose β = 4 (scale-free R^2^ = 0.85) to construct a scale-free network (Fig. 3C). The modules with similarities > 0.75 were merged, and 12 modules were retrieved (Fig. 3D). Figure 3E illustrates the correlation between each module and immune traits. We found that Th1 cells were highly correlated with blue modules (Cor = 0.942, *P* = 1.57E-06). CD56bright NK cells were highly correlated with turquoise module (Cor = 0.917, *P* = 1.05E-05). Neutrophils were highly correlated with the turquoise module (Cor = 0.693, *P* = 0.008). Numerous studies have shown that the immune response mediated by Th1 cells is closely related to NP. To clarify the potential role and mechanism of Th1 cells in NP, we selected the blue module with the highest correlation with Th1 cells as the core module for subsequent analysis. According to the screening criteria, GS > 0.5 and MM > 0.8, 305 genes were identified in the blue module (Fig. 3F). Next, we constructed a Venn plot to show the intersection genes of the core module and DEGs (Fig. 3G). These intersection genes, including *Abca1*, *Bach1*, *Cckbr*, *Col5a3*, and *Fyb*, were selected as the key candidates.

### Identifying and verifying key genes

**Fig. 4:**
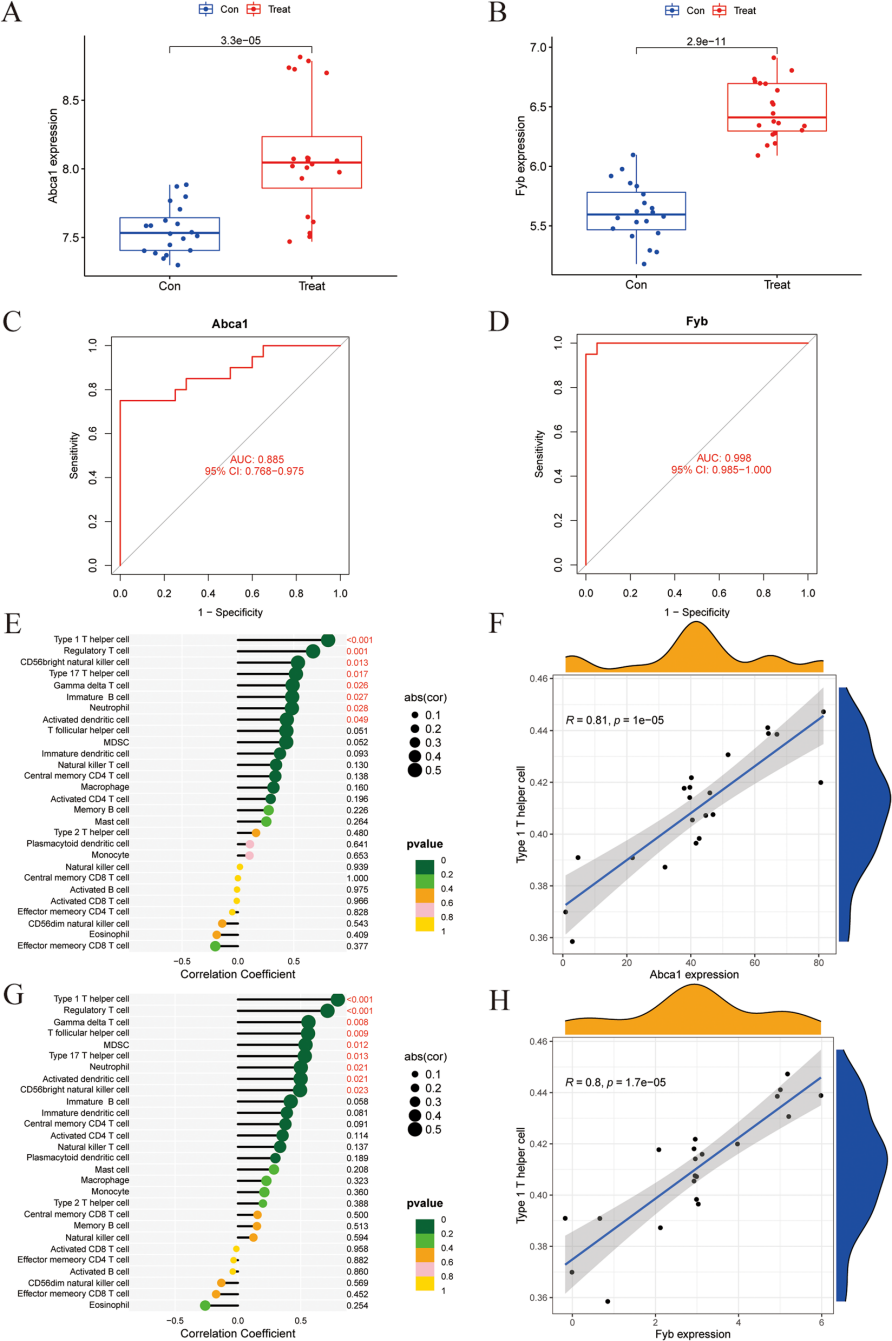
Verification the correlation between Hub gene expression and immunity. **A**, **B** Hub gene expression level was verified by external dataset; (**C**, **D**) ROC curve shows validation efficiency; (E) Lollipop plot showed the correlation between Abca1 and infiltrating immune cells. (The larger the circle size, the stronger the correlation; *p* < 0.05 was considered statistically significant.); (**F**) The correlation between Abca1 and Th1 cells. **G** Lollipop plot showed the correlation between Fyb and infiltrating immune cells. (The larger the circle size, the stronger the correlation; *p* < 0.05 was considered statistically significant.); (**H**) The correlation between Fyb and Th1 cells

The expression of the candidate key genes was verified using an external dataset (GSE24982). Figures 4A and B show that *Abca1* and *Fyb* were significantly upregulated in the SNI group of the GSE24982 dataset, and the receiver operating characteristic (ROC) curves were drawn to evaluate the verification reliability (Figs. 4C and D). However, it was found that Bach1 did not exist in the GSE24982 dataset, while Cckbr and Col5a3 did not differ significantly between the experimental and the control group. Therefore, follow-up studies mainly focused on Abca1 and Fyb. Eventually, *Abca1* [area under the curve (AUC) = 0.885, 95% confidence interval (CI): 0.768–0.975] and *Fyb* (AUC = 0.998, 95% CI: 0.985–1.000) were identified as the key genes for Th1 cell infiltration in DRG. Subsequently, Spearman’s correlation analysis was performed to verify the correlation between *Abca1*, *Fyb* and immune cell infiltration. The results showed a correlation between Abca1 and Th1 T cells, regulatory T cells, CD56bright NK cells, type 17 Th cells, gamma delta T cells, immature B cells, neutrophils, and activated dendritic cells was statistically significant (Fig. 4E). Notably, Abca1 was significantly correlated with Th1 cells (*R* = 0.81, *P* = 1E-05) (Fig. 4F). Moreover, the correlation between Fyb and Th1 cells, regulatory T cells, gamma delta T cells, myeloid-derived suppressor cells (MDSCs), type 17 Th cells, neutrophils, activated dendritic cells, and CD56bright NK cells was statistically significant (Fig. 4G). Especially, Fyb was significantly correlated with Th1 cells (*R* = 0.8, *P* = 1.7E-05) (Fig. 4H).

### scRNA-Seq data revealed high cellular heterogeneity in DRG tissues

**Fig. 5:**
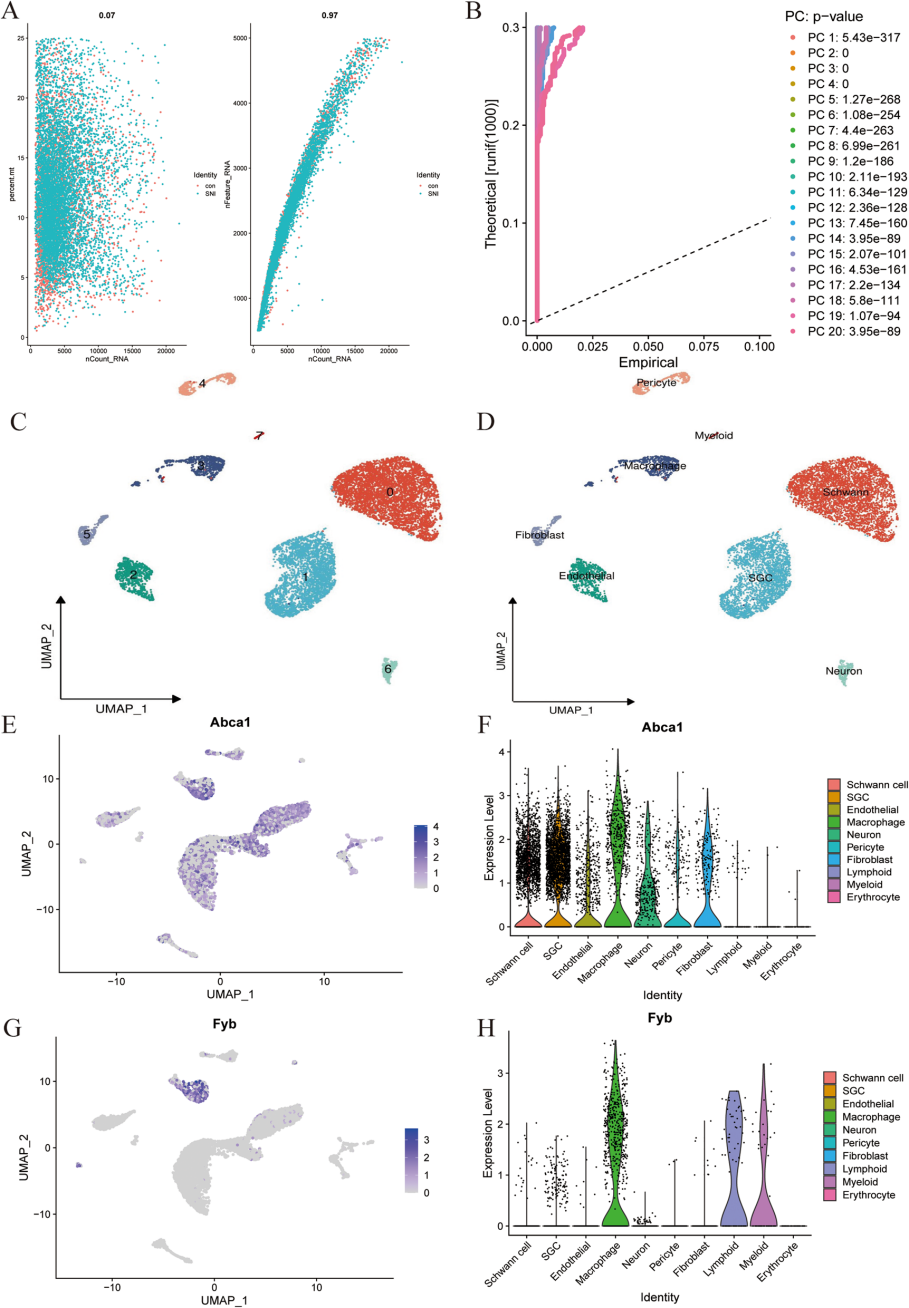
Flow analysis of single cell data and expression pattern of Hub gene at single cell level. **A**, **B** Gene filtering and PCA clustering of the gene expression matrix. **C**, **D** t-SNE projections and cell subset annotation of SNI model DRG tissue. **E**, **F** Expression pattern of Abca1 at the single cell level, shown in t-SNE figures and violin maps. (G, H) Expression pattern of Fyb at the single cell level, shown in t-SNE figures and violin maps

We downloaded mouse scRNA sequencing data from Sham and SNI models to determine the single-cell level pattern in DRG tissues. First, we performed quality control on the gene expression matrix (Fig. 5A). Then, the RNA-seq data were normalized, and 15 PCs (*P* < 0.05) were screened for subsequent analysis (Fig. 5B). Then t-distributed random neighborhood embedding (t-SNE) was used for unsupervised cell clustering analysis (Fig. 5C). Figure 5D shows that DRG tissue is isolated into nine distinct clusters, including schwann, SGC, endothelial, macrophage, pericyte, fibroblast, neuron, and myeloid. Next, we tested the expression patterns of candidate key genes in these cell clusters and verified their expression patterns at the single-cell level. As expected, they were significantly expressed in immune-related cells; the key gene *Abca1* (Figs. 5E and F) and *Fyb* (Figs. 5G and H) was highly expressed in macrophages.

### Abca1 was highly expressed in mouse DRG tissue and significantly affected M1 polarization of macrophages

**Fig. 6:**
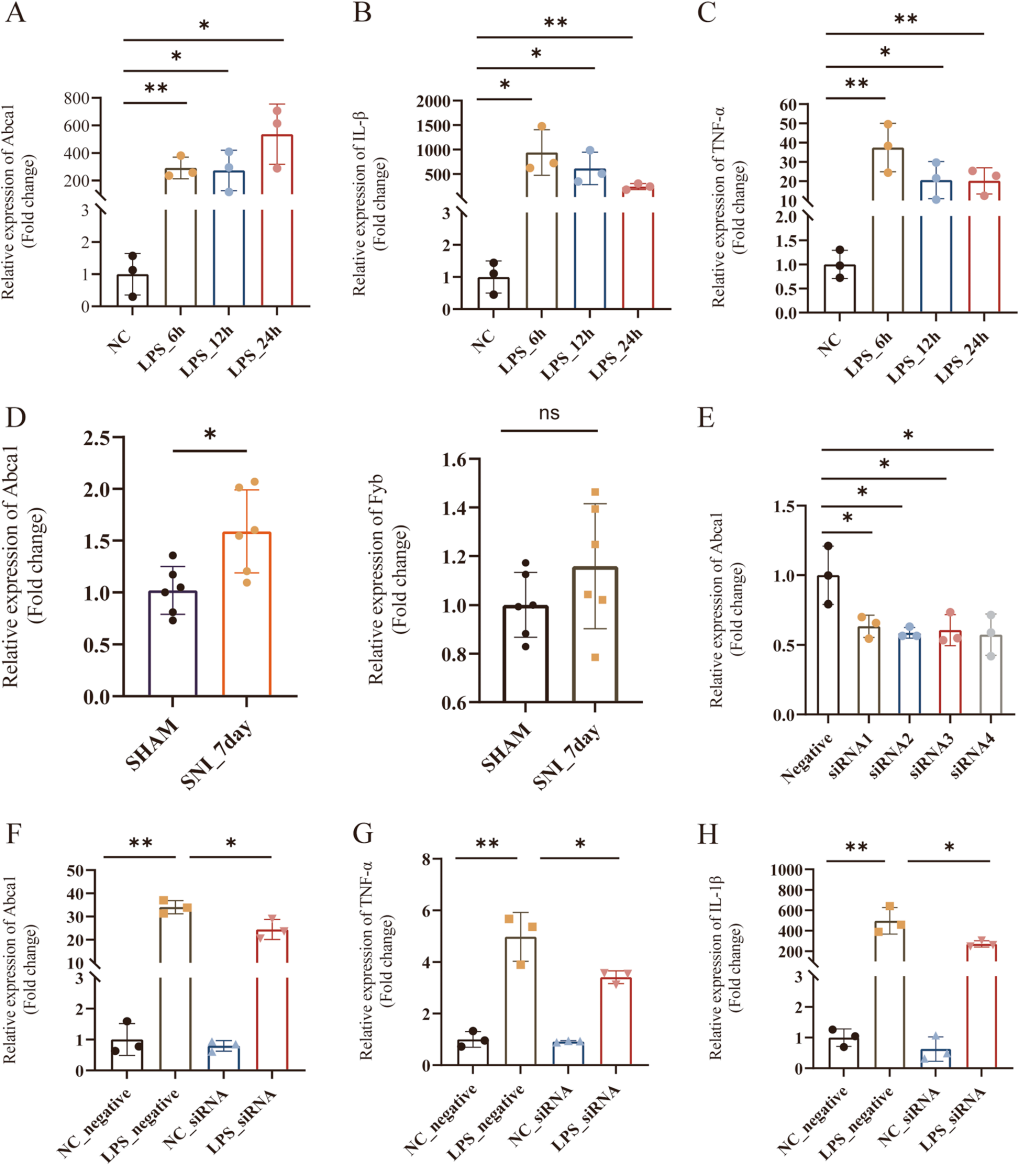
In vivo and in vitro experimental verification. **A**, **B**, **C** Expression levels of Abca1 and inflammatory factors in Raw264.7 cells stimulated by (LPS 1µg/mL) at different times. **D** Expression levels of Abca1 and Fyb in SNI model DRG tissue. **E** Abca1 silencing efficiency. **F**, **G**, **H** Expression levels of inflammatory factors after Abca1 was down-regulated

The expression of *Abca1* was further validated in animal and cell experiments. RAW264.7 cells were stimulated with 1µg/mL LPS at different time points to detect Abca1 and the corresponding inflammatory indicators (Fig. 6A). As expected, the results showed that *Abca1* expression was significant at 6 h after LPS stimulation, and the inflammatory factors, TNF-α and IL-1β of macrophage M1 polarization were significantly increased (Figs. 6B and C). Subsequently, qRT-PCR results (Fig. 6D) showed that *Abca1* expression level increased significantly in SNI model DRG tissues. However, there was no significant difference in the expression level of Fyb (Fig. 6D). Therefore, our subsequent studies mainly focus on Abca1. To further explore the effect of Abca1 on macrophage function, we knocked down Abca1 in macrophages. Figure 6E shows the siRNA knockdown efficiency; finally, siRNA4 was selected for subsequent assays. The results showed that when Abca1 expression was downregulated, the expression of macrophage inflammatory factors, TNF-α, and IL-1β was decreased (Fig. 6F, G, and H). Therefore, we speculated that Abca1 affects the polarization function of macrophage M1.

## Discussion

The pathogenesis of NP is complex, but effective clinical treatment is lacking [14]. The interference strategies of immune cell molecular function and signaling have shown great therapeutic potential in many diseases, yet their mechanism of action in NP is poorly understood [15, 16]. The present study aimed to analyze the patterns of immune cell infiltration in DRG tissues in NP and identify the key biomarkers. T cells act on intracellular signal transduction pathways by secreting various cytokines and expressing multiple transcription factors to participate in the occurrence and development of NP [17–19]. Subsequently, six hub genes (*Abca1*, *Bach1*, *Cckbr*, *Col5a3*, and *Fyb*) were closely related to Th1 cells infiltration, indicating their role in the occurrence and progression of NP. Among the candidate Th1 infiltration-related genes, *Abca1* was validated as a potential key biomarker and target of NP. Novel hub genes and pattern recognition of immune infiltration have expanded the understanding of NP pathogenesis.

In this study, GO analysis (Figs. 2E) showed that the SNI group was significantly enriched in glucocorticoid metabolism, synaptic component composition, G protein-coupled peptide receptor activity, and fatty acid linkage compared to the control group. Although glucocorticoids are classic anti-inflammatory drugs, activation of central glucocorticoid receptors might aggravate hippocampal neuronal death and increase the neurotoxicity of CNS inflammation [20, 21]. In addition, previous studies have shown that inflammatory cytokines regulate synaptic structure and function after peripheral nerve injury, which might underlie NP and memory deficits caused by peripheral nerve injury [18, 19]. Jiang et al. [22] demonstrated that GPR151, a Gβγ-coupled receptor, induces ERK-dependent neuroinflammatory response and is involved in the maintenance of trigeminal neuralgia. Therefore, GPR151 may be a potential drug target for treating trigeminal neuralgia. Furthermore, a recent study showed that ω-6 polyunsaturated fatty acids exacerbate preclinical inflammation and induce reversible peripheral nerve dysfunction resulting in pain [23]. Moreover, KEGG analysis (Figs. 2F) showed that neuroactive ligand-receptor effects [24], calcium signaling pathway [25], PI3K-Akt signaling pathway [26], MAPK signaling pathway [27], and TGF-β signaling pathway differ significantly between the SNI and control groups. These signaling pathways have been studied extensively with respect to inflammatory effects and NP. In conclusion, the GO and KEGG results support the role of inflammation in NP.

In this study, the infiltration level of Th1 cells was significantly higher in the SNI group than in the Con group in DRG tissue. Reportedly, CD4 T lymphocytes (mainly Th1 cells) infiltrating the spinal cord develop nerve injury-induced behavioral hypersensitivity responses [28]. WGCNA indicated that the blue module was enriched for genes expressed during Th1 cell infiltration. Furthermore, the intersection genes of the blue module and DEGs were considered hub genes related to Th1 infiltration (*Abca1*, *Bach1*, *Cckbr*, *Col5a3*, and *Fyb*). Finally, we identified *Abca1* and *Fyb* as the key genes closely associated with Th1 cell infiltration in NP pain through external data set validation. Correlation analysis between *Abca1* and immune cells showed that *Abca1* was significantly correlated with Th1 cells (*R* = 0.81, *P* = 1E-05). Interestingly, single-cell analysis revealed that Th1 infiltration-related genes (*Abca1* and *Fyb*) were expressed in macrophages. Reportedly, the non-neuronal interactions in DRG tissue influence the development of NP [29]. Davoli-Ferreira et al*.* [30] demonstrated that regulatory T cells relieve NP by inhibiting the Th1 response at the site of peripheral nerve injury. Although the results of immune infiltration showed no significant difference in the infiltration level of macrophages between the SNI and Con groups, macrophages have extremely high plasticity and complex heterogeneity, so it is essential to understand the molecular characteristics of macrophages in different times and spaces and different lineages. Studies have shown that there are two sources of macrophages: macrophages (MDMs) derived from monocytes; the other are macrophages (TRMs) that reside in tissues [31]. *Miriam Merad* [32] study found that TRMs highly expressed Ccl17 and Tgfb1 and proved that these genes could promote the recruitment, differentiation, and amplification of Treg cells, resulting in a significant increase in the number of regulatory T (Treg) cells in tissues. Undoubtedly, it is substantial to trace the origin of macrophages with high Abca1 expression and explore how it affects Th1 cell infiltration. Therefore, we hypothesized that *Abca1* promotes Th1 cells infiltration by affecting macrophage function in DRG tissues, thereby inducing NP.

*Abca1* is a member of the ATP-binding cassette transporter (ABC) superfamily and a crucial cell-surface protein promoting cholesterol efflux. Glial cells express TLR4, mediating the secretion of inflammatory cytokines, chemokines, and bioactive lipids [33, 34]. Neuroinflammation mediated by infiltrating immune cells in the spinal cord and DRGs is an essential component of neuropathy [35]. However, the mechanism by which Th1 cells infiltration in DRG tissue induces abnormal pain after peripheral nerve injury has not been studied extensively. The current results showed that the expression level of *Abca1* was significantly increased in DRG tissues of the SNI model. Subsequently, after stimulation of RAW264.7 cells with 1 g/mL LPS, the levels of TNF-α and IL-1β were increased significantly; however, this effect was reversed when *Abca1* was knocked down in macrophages. Therefore, we proposed that *Abca1* affects the function of M1 polarization in macrophages. Niehaus et al. [36] found that macrophages in DRG are critical in initiating and maintaining mechanical hypersensitivity in NP in mice. Furthermore, interferon-gamma (IFN-γ) is a potent macrophage activator [37], and thus, the mechanism underlying the interaction between macrophages, Th1 cells and inflammatory effect in DRG leading to NP needs an in-depth exploration. Therefore, the underlying mechanism may be that *Abca1* induces M1 activation of macrophages to secrete proinflammatory factors, which in turn promotes naive CD4 T cells to differentiate into Th1 cells and enhances Th1 cell-derived IFN-γ-mediated inflammatory effects.

Reportedly, T cells infiltrate the damaged sciatic nerve after CCI, and passive transfer of Th1 T cells can restore the nerve sensitivity in athymic nude mice [38]. Costigan et al*.* [10] demonstrated a functional role of IFN-γ signaling in generating pain-like hypersensitivity responses after nerve injury in adults. Intrathecal injection of IFN-γ into the spinal cord induces hyperalgesia in naive animals [39], stimulates microglia via IFN-γR, interferes with the signaling, and inhibits neural mechanical hypersensitivity [40]. Despite the close association of Th1 cytokines with nerve injury-induced pain, the exact contribution of Th1 cells remains to be determined. An in-depth study of immune cell crosstalk would clarify the specific role of infiltrating CD4 T lymphocytes in developing NP and guide the designing therapies for the cytokines/mechanisms underlying NP. Therefore, intervention strategies targeting Th1 cells infiltration into the DRG might contribute to the establishment of anti-NP hypersensitivity responses in humans.

**Fig. 7:**
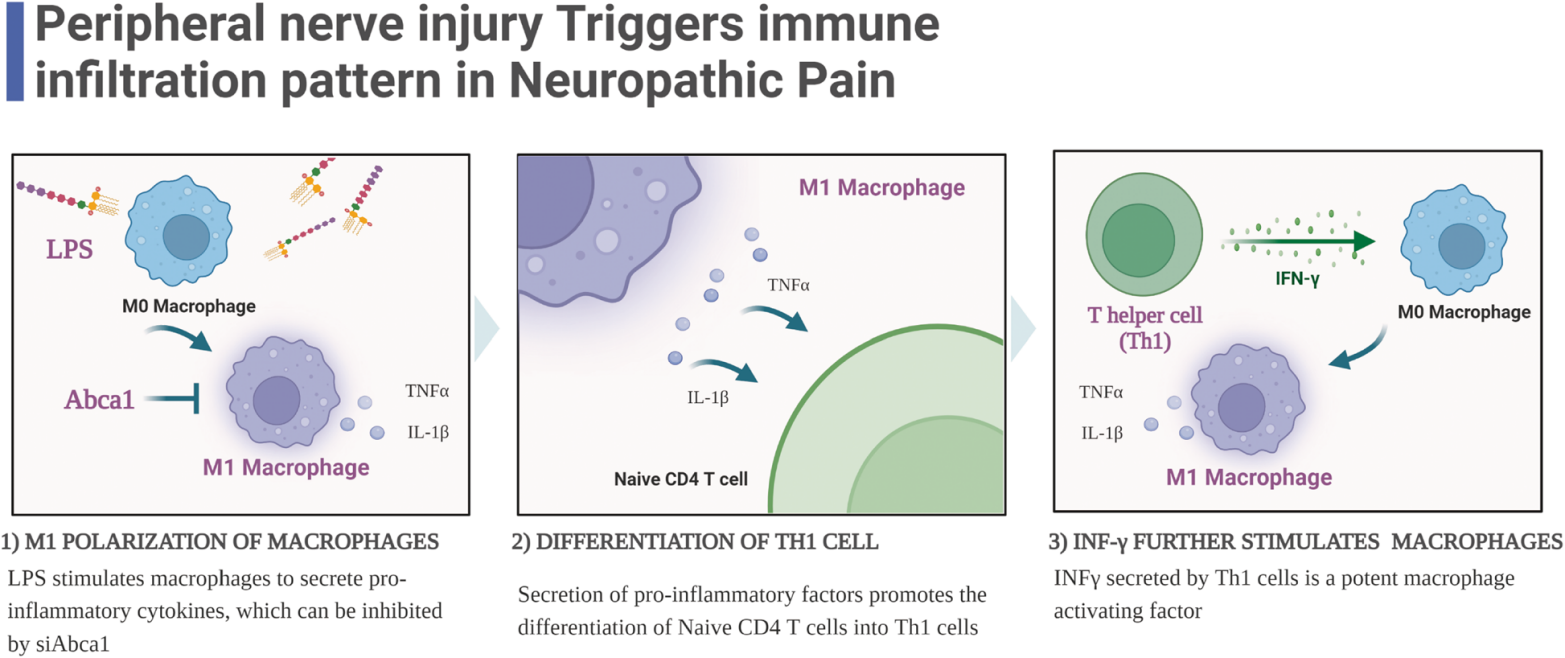
Hypothesis mechanism diagram (Created with BioRender.com)

Neuroimmune signaling might underlie the development of abnormal pain after nerve injury [41, 42]. Our study showed that Abca1 siRNA treatment reduced the mRNA levels of proinflammatory mediators, including *TNF-α*, and *IL-1β*, in cultured RAW264.7 cells activated by LPS. Interestingly, Abca1 was closely associated with Th1 cell infiltration in the model of peripheral nerve injury. Therefore, the effect of Abca1 on macrophage function and mediating Th1 cell infiltration will further expand Neuroimmune signaling communication in neuropathic pain; the underlying mechanism is shown in Fig. 7. Developing targeted therapies for peripheral NP is essential by using immune infiltration-related genes as starting points.

## Materials and Methods

### Data acquisition and preprocessing

The bulk datasets (GSE102721, GSE149770, and GSE24982) and single-cell transcriptome data (GSE174430) were obtained from Gene Expression Omnibus (GEO) database (https://www.ncbi.nlm.nih.gov/geo/). First, the practical extraction and reporting language (Perl) (https://www.perl.org/get.html) was used for quick and accurate processing of the file paths that required R package analysis. Then, R language (Rv3.6.3 and Rv4.1.1) (https://www.r-project.org/) was used for data analysis. Next, two gene expression matrices were merged using the “limma” package [43], and batch effects were removed for subsequent analysis. Finally, the “ggplot2” package [44] was used to draw UMAP to visualize the results of batch effect removal.

### Difference and enrichment analysis

The “limma” package was applied to screen the differentially expressed genes (DEGs) in the Sham and SNI groups. *P*-value < 0.05 and |log2 FC|> 0.5 defined the significantly different genes. The “ggplot2” package drew the heat map and the volcano plot. The “clusterProfiler” [45], “org.Mm.eg.db”, and “ggplot2” packages were applied for the analysis of Gene Ontology (GO) [46] and Kyoto Encyclopedia of Genes and Genomes (KEGG) [47, 48]. The GO analysis included biological process (BP), molecular function (MF), and cellular component (CC). KEGG analysis was used to determine the pathways of biological molecular interaction. A false-discovery rate (FDR) < 0.25 and *P* < 0.05 were used to screen out significant functional enrichment.

### Evaluation of immune cell infiltration

The ssGESA algorithm was applied to explore the level of immune cell infiltration using packages “GSVA” [49] and “GSEABase.” In addition, the “ggplot2” package was applied to draw a boxplot and illustrate the differences in infiltrating immune cells. Furthermore, 28 types of infiltrating immune cells were plotted using the “corrplot” package (https://github.com/taiyun/corrplot).

### WGCNA

WGCNA [13] was employed to identify the immune cell infiltration-related module genes and explore the correlation between the immunophenotypes and module genes in the network. The top 5000 genes with median absolute deviation were screened out for subsequent analysis. First, the outliers of gene expression matrices were filtered by hierarchical cluster analysis. β = 4 (scale-free R^2^ = 0.85) was selected to construct a scale-free network. Then, a dynamic hybrid cutting technology was used to construct the gene modules, and a cluster dendrogram was drawn (minModuleSize = 50, mergeCutHeight = 0.25, the colors represent different modules). Next, the correlation heatmap between modules and immune traits was drawn; rows represented modules, columns represented traits, and the correlation and *P*-value were displayed in boxes. The correlation between gene expression and immune characteristics was determined by screening gene significance (GS > 0.5) and module membership (MM > 0.8). Finally, the intersection of genes of interesting modules and DEGs constituted the candidate key genes. The “VennDiagram” package [50] was used to draw the Venn plot and show the intersection results.

### Identification and verification of key genes

The differential expression of candidate key genes was verified through an external dataset, and the key genes with the highest significance were determined. Next, the “ggpubr” and “ggExtra” packages were applied for Spearman’s correlation analysis on key genes and infiltrating immune cells, and a lollipop plot was used to visualize the analysis results.

### Analysis at the single-cell level

DRG tissue-derived single-cell RNA sequencing dataset in the SNI model (GSE174430) was downloaded from the GEO database. “Seurat” [51] package was used for downstream analysis. The cells were filtered with nFeature_RNA > 500 and nFeature_RNA < 5000 and percent.mt < 5%. “LogNormalize” method was utilized to normalize and scale gene expression. Then, PCA was used to identify the major principal components (PCs), and the JackStraw and ScoreJackStraw functions were used to visualize the P-value distribution. “Harmony” package was used for batch correction to avoid batch effects, and the FindClusters function was used to classify the cells into eight clusters with a resolution of 0.1. The cell types were manually annotated by cellMarker and panglaoDB. Finally, the expression patterns of key genes were identified and visualized by t-SNE and VlnPlot.

### Animals and models

A total of 12 healthy 8-week-old male mice were purchased from the Experimental Animal Center of Guangzhou university of Chinese Medicine and randomly and equally divided into two groups (Sham and SNI). Before any experiment, all animals were acclimatized for one week. First, SNI model [52] was established, and 10% chloral hydrate (0.3 mL/100 g) was injected intraperitoneally for anesthesia. The sciatic nerve and its branches were then exposed. Next, the tibial and the common peroneal nerve were ligated with 6–0 non-invasive sutures and closely connected with distal resection, leaving the sural nerve intact. In the Sham group, the sciatic nerve and its branches were exposed but not ligated.

### Cell culture and inflammatory model

Mouse macrophage cell line RAW264.7 was cultured in DMEM/High Glucose with 10% fetal bovine serum (FBS), 100 U/mL of penicillin, and 100 µg/mL of streptomycin and maintained at 37 °C in the atmosphere of 95% humidity and 5% CO_2_. RAW 264.7 cells were plated at a density of 1 × l0^5^ cells/mL/ well in 6-well plates and divided into four groups. Lipopolysaccharide (LPS) was solubilized in phosphate-buffered saline (PBS). The control group was treated only with DMEM maintenance medium. The LPS group was stimulated at 6, 12, and 24 h, respectively, and the optimal time points were selected for subsequent studies.

### Identification of efficient Abca1 siRNA

The siRNA of Abca1 was designed and synthesized by Tsingke Biotechnology (Beijing, China), and the sequence was as follows: siAbca1-Mouse (#1): 5’-UUGAUGAGCCUGACUUCUGTT-3’ and 5’-CAGAAGUCAGGCUCAUCAATT-3’; siAbca1-Mouse (#2): 5’-UAGUUGUUAUCCUCGUACCTT-3’ and 5’-GGUACGAGGAUAACAACUATT-3’; siAbca1-Mouse (#3): 5’-AGUAGAUCUUGGAAGGGACTT-3’ and 5’-GUCCCUUCCAAGAUCUACUTT-3’; siAbca1-Mouse (#4): 5’-AUGACAACCUUGGAUCCACTT-3’ and 5’-GUGGAUCCAAGGUUGUCAUTT-3’. RAW 264.7 cells were plated at a density of 1 × l0^5^ cells/mL/well in 6-well plates and divided into five groups. According to the manufacturer's instructions, when the cells had grown to 60–70%, the cells were transfected using the ZetaLife Advanced DNA/RNA Transfection reagent. Total RNA of cells was extracted to detect the expression of *Abca1*, and siRNA with the highest knockdown efficiency was screened out for subsequent studies.

### RNA extraction and quantitative real-time polymerase chain reaction (qRT-PCR)

Trizol reagen (Beyotime, R0016) was used to extract total RNA from cells and DRG tissue. Reverse transcription was performed using Prime Script RT reagent kit (Perfect Real Time). β-actin was used as an internal reference. RT-qPCR assays were carried out using Light Cycle instrument, and the relative expression level was analyzed using the 2^-(∆∆Ct) formula. The primer sequences are listed in Table 2.

**Table 2:** Primers used for qRT-PCR

Gene name	Forward primer(5'⁃3')	Reverse primer(5'⁃3')
β-actin	AGGGAAATCGTGCGTGACAT	GAACCGCTCATTGCCGATAG
Abca1	TTAAAAACCTGGATCGGAACCAA	GCATTAGCTTCAGATTTACGGGT
TNF-α	AGGTTCTGTCCCTTTCACTCACTGG	AGAGAACCTGGGAGTAGACAAGGTA
IL-1β	TGGCAATGAGGATGACTTGT	GTGGTGGTCGGAGATTCGTA

### Statistical analysis

All statistical analyses were conducted in the R language (Rv3.6.3 and Rv4.1.1). All statistical tests were bilateral, and *P* < 0.05 indicated a statistically significant difference.

## Conclusion

Herein, whole transcriptome and single-cell sequencing data were combined to investigate the pattern of immune infiltration in DRG tissues and the key genes in NP. The current study showed that Th1 infiltration in DRG tissue was involved in the pathological process of NP induced by peripheral nerve injury. The underlying mechanism may be that *Abca1* affects the M1 polarization of macrophages and promotes Th1 cell infiltration in DRG tissues. However, the mechanism of neuroimmune inflammation is rather complex and needs further investigation. This study provided novel ideas and potential targets for the pathogenesis and treatment of NP.

## Data Availability

Original contributions are included in the article/supplementary materials. Publicly available datasets can be found here: https://www.ncbi.nlm.nih.gov/geo/query/acc.cgi?acc=GSE102721 https://www.ncbi.nlm.nih.gov/geo/query/acc.cgi?acc=GSE149770 https://www.ncbi.nlm.nih.gov/geo/query/acc.cgi?acc=GSE24982 https://www.ncbi.nlm.nih.gov/geo/query/acc.cgi?acc=GSE174430
